# ODYSSEY clinical trial design: a randomised global study to evaluate the efficacy and safety of dolutegravir-based antiretroviral therapy in HIV-positive children, with nested pharmacokinetic sub-studies to evaluate pragmatic WHO-weight-band based dolutegravir dosing

**DOI:** 10.1186/s12879-020-05672-6

**Published:** 2021-01-04

**Authors:** Cecilia L. Moore, Anna Turkova, Hilda Mujuru, Adeodata Kekitiinwa, Abbas Lugemwa, Cissy M. Kityo, Linda N. Barlow-Mosha, Tim R. Cressey, Avy Violari, Ebrahim Variava, Mark F. Cotton, Moherndran Archary, Alexandra Compagnucci, Thanyawee Puthanakit, Osee Behuhuma, Yacine Saϊdi, James Hakim, Pauline Amuge, Lorna Atwine, Victor Musiime, David M. Burger, Clare Shakeshaft, Carlo Giaquinto, Pablo Rojo, Diana M. Gibb, Deborah Ford, the ODYSSEY Trial Team, Shabinah Ali, Abdel Babiker, Chiara Borg, Anne-Marie Borges Da Silva, Joanna Calvert, Deborah Ford, Joshua Gasa, Diana M. Gibb, Nasir Jamil, Sarah Lensen, Emma Little, Fatima Mohamed, Samuel Montero, Cecilia L. Moore, Rachel Oguntimehin, Anna Parker, Reena Patel, Tasmin Phillips, Tatiana Sarfati, Karen Scott, Clare Shakeshaft, Moira Spyer, Margaret Thomason, Anna Turkova, Rebecca Turner, Nadine Van Looy, Ellen White, Kaya Widuch, Helen Wilkes, Ben Wynne, Carlo Giaquinto, Tiziana Grossele, Daniel Gomez-Pena, Davide Bilardi, Giulio Vecchia, Alexandra Compagnucci, Yacine Saidi, Yoann Riault, Alexandra Coelho, Laura Picault, Christelle Kouakam, Tim R. Cressey, Suwalai Chalermpantmetagul, Dujrudee Chinwong, Gonzague Jourdain, Rukchanok Peongjakta, Pra-ornsuda Sukrakanchana, Wasna Sirirungsi, Janet Seeley, Sarah Bernays, Magda Conway, Nigel Klein, Eleni Nastouli, Anita De Rossi, Maria Angeles Munoz Fernandez, David Burger, Pauline Bollen, Angela Colbers, Hylke Waalewijn, Cissy M. Kityo, Victor Musiime, Elizabeth Kaudha, Annet Nanduudu, Emmanuel Mujyambere, Paul Ocitti Labeja, Charity Nankunda, Juliet Ategeka, Peter Erim, Collin Makanga, Esther Nambi, Abbas Lugemwa, Lorna Atwine, Edridah Keminyeto, Deogratiuos Tukwasibwe, Shafic Makumbi, Emily Ninsiima, Mercy Tukamushaba, Rogers Ankunda, Ian Natuhurira, Miriam Kasozi, Baker Rubinga, Adeodata R. Kekitiinwa, Pauline Amuge, Dickson Bbuye, Justine Nalubwama, Winnie Akobye, Muzamil Nsibuka Kisekka, Anthony Kirabira, Gloria Ninsiima, Sylvia Namanda, Gerald Agaba, Immaculate Nagawa, Annet Nalugo, Florence Namuli, Rose Kadhuba, Rachael Namuddu, Lameck Kiyimba, Angella Baita, Eunice Atim, Olivia Kobusingye, Clementine Namajja, Africanus Byaruhanga, Rogers Besigye, Herbert Murungi, Geoffrey Onen, Philippa Musoke, Linda Barlow-Mosha, Grace Ahimbisibwe, Rose Namwanje, Monica Etima, Mark Ssenyonga, Robert Serunjogi, Hajira Kataike, Richard Isabirye, David Balamusani, Monica Nolan, Mark F. Cotton, Anita Janese van Rensburg, Marlize Smuts, Catherine Andrea, Sumaya Dadan Sonja Pieterse, Vinesh Jaeven, Candice Makola, George Fourie, Kurt Smith, Els Dobbels, Peter Zuidewind, Hesti Van Huyssteen, Mornay Isaacs, Georgina Nentsa, Thabis Ncgaba, Candice MacDonald, Mandisa Mtshagi, Maria Bester, Wilma Orange, Ronelle Arendze, Mark Mulder, George Fourie, Avy Violari, Nastassja Ramsagar, Afaaf Liberty, Ruth Mathiba, Lindiwe Maseko, Nakata Kekane, Busi Khumlo, Mirriam Khunene, Noshalaza Sbisi, Jackie Brown, Ryphina Madonsela, Nokuthula Mbadaliga, Zaakirah Essack, Reshma Lakha, Aasia Vadee, Derusha Frank, Nazim Akoojee, Maletsatsi Monametsi, Gladness Machache, Yolandie Fourie, Anusha Nanan-kanjee, Juan Erasmus, Angelous Mamiane, Tseleng Daniel, Fatima Mayat, Nomfundo Maduna, Patsy Baliram, Chaiwat Ngampiyasakul, Pisut Greetanukroh, Wanna Chamjamrat, Praechadaporn Khannak, Pornchai Techakunakorn, Thitiwat Thapwai, Patcharee Puangmalai, Ampai Maneekaew, Pradthana Ounchanum, Yupawan Thaweesombat, Areerat Kongponoi, Jutarat Thewsoongnoen, Suparat Kanjanavanit, Pacharaporn Yingyong, Thida Namwong, Rangwit Junkaew, Ussanee Srirompotong, Patamawadee Sudsaard, Siripun Nuanbuddee, Sookpanee Wimonklang, Sathaporn Na-Rajsima, Suchart Thongpaen, Pattira Runarassamee, Watchara Meethaisong, Arttasid Udomvised, Ebrahim Variava, Modiehi Rakgokong, Dihedile Scheppers, Tumelo Moloantoa, Abdul Hamid Kaka, Tshepiso Masienyane, Akshmi Ori, Kgosimang Mmolawa, Pattamukkil Abraham, Moherndran Archary, Rejoice Mosia, Sajeeda Mawlana, Rosie Mngqibisa, Rashina Nundlal, Elishka Singh, Penelope Madlala, Allemah Naidoo, Sphiwee Cebekhulu, Petronelle Casey, Collin Pillay, Subashinie Sidhoo, Minenhle Chikowore, Lungile Nyantsa, Melisha Nunkoo, Terence Nair, Enbavani Pillay, Sheleika Singh, Sheroma Rajkumar, Osee Behuhuma, Olivier Koole, Kristien Bird, Nomzamo Buthelezi, Mumsy Mthethwa, James Hakim, Hilda Mujuru, Kusum Nathoo, Mutsa Bwakura-Dangarembizi, Ennie Chidziva, Shepherd Mudzingwa, Themelihle Bafana, Colin Warambwa, Godfrey Musoro, Gloria Tinago, Shirley Mutsai, Columbus Moyo, Ruth Nhema, Misheck Nkalo Phiri, Stuart Chitongo, Joshua Choga, Joyline Bhiri, Wilber Ishemunyoro, Makhosonke Ndlovu, Thanyawee Puthanakit, Naruporn Kasipong, Sararut Chanthaburanun, Kesdao Nanthapisal, Thidarat Jupimai, Thornthun Noppakaorattanamanee, Torsak Bunupuradah, Wipaporn Natalie Songtaweesin, Chutima Saisaengjan, Stephan Schultze-Straber, Christoph Konigs, Robin Kobbe, Felicia Mantkowski, Steve Welch, Jacqui Daglish, Laura Thrasyvoulou, Delane Singadia, Sophie Foxall, Judith Acero, Gosia Pasko-Szcech, Jacquie Flynn, Gareth Tudor-Williams, Farhana Abdulla, Srini Bandi, Jin Li, Sean O’Riordan, Dominique Barker, Richard Vowden, Colin Ball Eniola Nsirim, Kathleen McClughlin, India Garcia, Pablo Rojo Conejo, Cristina Epalza, Luis Prieto Tato, Maite Fernandez, Luis Escosa Garcia, Maria José Mellado Peña, Talia Sainz Costa, Claudia Fortuny Guasch, Antoni Noguera Julian, Carolina Estepa, Elena Bruno, Alba Murciano Cabeza, Maria Angeles Muñoz Fernandez, Paula Palau, Laura Marques, Carla Teixeira, Alexandre Fernandes, Rosita Nunes, Helena Nascimento, Andreia Padrao, Joana Tuna, Helena Ramos, Ana Constança Mendes, Helena Pinheiro, Ana Cristina Matos, Flavia Kyomuhendo, Sarah Nakalanzi, Cynthia Mukisa Williams, Ntombenhle Ngcobo, Deborah Pako, Jacky Crisp, Benedictor Dube, Precious Chandiwana, Winnie Gozhora, Ian Weller, Elaine Abrams, Tsitsi Apollo, Polly Clayden, Valériane Leroy, Anton Pozniak, Jane Crawley, Rodolphe Thiébaut, Helen McIlleron, Alasdair Bamford, Hermione Lyall, Andrew Prendergast, Felicity Fitzgerald, Anna Goodman

**Affiliations:** 1 Medical Research Council Clinical Trials Unit at University College London, London, United Kingdom; 2 University of Zimbabwe Clinical Research Centre, Harare, Zimbabwe; 3 Baylor College of Medicine Children’s Foundation, Kampala, Uganda; 4 Joint Clinical Research Centre, Mbarara, Uganda; 5 Joint Clinical Research Centre, Kampala, Uganda; 6 MUJHU Research Collaboration, Kampala, Uganda; 7 PHPT/IRD 174, Faculty of Associated Medical Sciences, Chiang Mai University, Chiang Mai, Thailand; 8 Department of Immunology & Infectious Diseases, Harvard T. H Chan School of Public Health, Boston, USA; 9 Department of Molecular & Clinical Pharmacology, University of Liverpool, Liverpool, UK; 10 Perinatal HIV Research Unit, Johannesburg, South Africa; 11 Klerksdorp Tshepong Hospital Complex, Matlosana, South Africa; 12 Family Center for Research with Ubuntu, Cape Town, South Africa; 13 Durban International Clinical Research Site, Durban, South Africa; 14 INSERM/ANRS SC10-US19, Paris, France; 15 HIVNAT, Thai Red Cross AIDS Research Center and Department of Pediatrics, Faculty of Medicine, Chulalongkorn University, Bangkok, Thailand; 16 Africa Health Research Institute, Hlabisa Hospital, Hlabisa, South Africa; 17 Department of Clinical Pharmacy and Nijmegen Institute for Infection, Inflammation and Immunity (N4i), Radboud University, Nijmegen, The Netherlands; 18 University of Padova, Padova, Italy; 19 Hospital 12 de Octubre, Madrid, Spain

**Keywords:** Randomized control trial, Basket trial, HIV, Paediatric, Efficacy, Safety, Pharmacokinetic, Dolutegravir

## Abstract

**Background:**

Dolutegravir (DTG)-based antiretroviral therapy (ART) is highly effective and well-tolerated in adults and is rapidly being adopted globally. We describe the design of the ODYSSEY trial which evaluates the efficacy and safety of DTG-based ART compared with standard-of-care in children and adolescents. The ODYSSEY trial includes nested pharmacokinetic (PK) sub-studies which evaluated pragmatic World Health Organization (WHO) weight-band-based DTG dosing and opened recruitment to children < 14 kg while dosing was in development.

**Methods:**

ODYSSEY (Once-daily DTG based ART in Young people vS. Standard thErapY) is an open-label, randomised, non-inferiority, basket trial comparing the efficacy and safety of DTG + 2 nucleos(t) ides (NRTIs) versus standard-of-care (SOC) in HIV-infected children < 18 years starting first-line ART (ODYSSEY A) or switching to second-line ART (ODYSSEY B). The primary endpoint is clinical or virological failure by 96 weeks.

**Results:**

Between September 2016 and June 2018, 707 children weighing ≥14 kg were enrolled; including 311 ART-naïve children and 396 children starting second-line. 47% of children were enrolled in Uganda, 21% Zimbabwe, 20% South Africa, 9% Thailand, 4% Europe. 362 (51%) participants were male; median age [range] at enrolment was 12.2 years [2.9–18.0]. 82 (12%) children weighed 14 to < 20 kg, 135 (19%) 20 to < 25 kg, 206 (29%) 25 to < 35 kg, 284 (40%) ≥35 kg. 128 (18%) had WHO stage 3 and 60 (8%) WHO stage 4 disease. Challenges encountered include: (i) running the trial across high- to low-income countries with differing frequencies of standard-of-care viral load monitoring; (ii) evaluating pragmatic DTG dosing in PK sub-studies alongside FDA- and EMA-approved dosing and subsequently transitioning participants to new recommended doses; (iii) delays in dosing information for children weighing 3 to < 14 kg and rapid recruitment of ART-naïve older/heavier children, which led to capping recruitment of participants weighing ≥35 kg in ODYSSEY A and extending recruitment (above 700) to allow for ≥60 additional children weighing between 3 to < 14 kg with associated PK; (iv) a safety alert associated with DTG use during pregnancy, which required a review of the safety plan for adolescent girls.

**Conclusions:**

By employing a basket design, to include ART-naïve and -experienced children, and nested PK sub-studies, the ODYSSEY trial efficiently evaluates multiple scientific questions regarding dosing and effectiveness of DTG-based ART in children.

**Trial registration:**

NCT, NCT02259127, registered 7th October 2014; EUDRACT, 2014–002632-14, registered 18th June 2014 (https://www.clinicaltrialsregister.eu/ctr-search/trial/2014-002632-14/ES); ISRCTN, ISRCTN91737921, registered 4th October 2014.

## Background

World Health Organization (WHO) guidelines recommend immediate ART for all adults and children living with HIV [1]. Although there has been substantial recent improvement in coverage of antiretroviral therapy (ART), only half of children in need have access to ART, with the majority (> 80%) living in Sub-Saharan Africa [2, 3]. The most commonly used regimens for human immunodeficiency virus (HIV)-infected children are triple combination ART with a ritonavir boosted protease inhibitor (bPI) or a non-nucleoside reverse transcriptase inhibitor (NNRTI). However, virological suppression on these regimens is worse compared to adults [4, 5], oral formulations of boosted-lopinavir are very poorly tolerated and there are concerns regarding pre-treatment resistance to NNRTIs in vertically-infected children [6, 7]. In this context, there is a need for alternative first- and second-line regimens for children with formulations which are well tolerated, easy for health workers to prescribe and for care-givers to administer.

Dolutegravir (DTG) is a second-generation integrase inhibitor with the advantage of once-daily dosing, a good short-term safety profile, low pharmacokinetic (PK) variability, few drug-to-drug interactions, rapid and robust virological response, a distinct resistance profile from raltegravir, a high genetic barrier to resistance and high potency at a low milligram dose. Following adult Phase II/III trials [8–12], the ODYSSEY trial aims to evaluate the efficacy and safety of once-daily DTG-based ART compared with standard-of-care (SOC) in children and adolescents (< 18 years) starting first- or second-line ART in resource-limited and well-resourced settings. We hypothesise that DTG with 2 nucleos(t) ides reverse transcriptase inhibitors (NRTIs) will be non-inferior to SOC in terms of efficacy and superior in terms of toxicity profile. This paper describes the design of the ODYSSEY trial, which has evolved over time to include nested PK sub-studies to evaluate pragmatic WHO weight-band-based DTG dosing and to open recruitment to children < 14 kg while dosing was still in development.

### Changing licensing and guidelines for Dolutegravir

When ODYSSEY opened in September 2016, the US Food and Drug Administration (FDA) and the European Medicines Agency (EMA) had licensed the adult DTG dose (50 mg film coated tablet (FCT)) in adolescents weighing ≥40 kg and the FDA had licensed DTG 35 mg FCT in children weighing between 30 and 40 kg. Additionally, data from the ongoing IMPAACT P1093, a paediatric Phase II/III dose-finding study, supported using DTG 25 mg FCT in children over 6 years weighing between 20 and 30 kg [13]. In February 2017, the EMA reduced the weight and age limits for children down to ≥15 kg and ≥ 6 years.

Supported by further emerging efficacy data in adults [14] and the low cost of DTG, a number of countries had procured DTG formulations for adults by the end of 2017 [4]. In May 2018, data from an unplanned analysis of a National Institutes of Health (NIH)-funded observational surveillance study of pregnancies in Botswana (TSEPAMO) suggested an increased risk of neural tube defects (NTD) in infants born to women who became pregnant while taking DTG (data from August 2014 through to March 2019 showed 5 cases of NTD in 1683 infants (0.3%), which compares to a risk of ~ 0.1% in infants born to women on non-DTG based regimens) [15–17]. WHO issued a statement advising of a potential DTG safety signal [18]. Despite this, in July 2018, in response to efficacy data and the increasing levels of pre-treatment ART resistance documented in low- and middle-income countries (LMICs), WHO updated its recommendations to include DTG-based ART as a preferred first-line regimen for HIV-positive individuals ≥6 years and ≥ 15 kg (with alternative options for females of child-bearing age not using effective contraception) [19]. Updated data from the TSEPAMO study suggested that any increased risk of NTDs is lower than originally reported [17, 20]. President’s Emergency Plan for AIDS Relief (PEPFAR) and other partners continue to encourage responsible rollout [21]. In their 2020 report, the Clinton Health Access Initiative (CHAI) predicted that DTG-based first-line regimens would have a 93% market share in LMICs by 2024 [21].

Paediatric DTG formulations lag behind the adult formulation significantly. Although paediatric film-coated formulations (10 mg FCT, 25 mg FCT) were approved by stringent regulatory authorities in the US and Europe in 2016–2017, they are still not available outside high resource countries. Paediatric dispersible tablets, having only been recently approved in the US, are not yet available in countries.

## Methods

### Study design, number of children, randomisation and follow-up

**Fig. 1:**
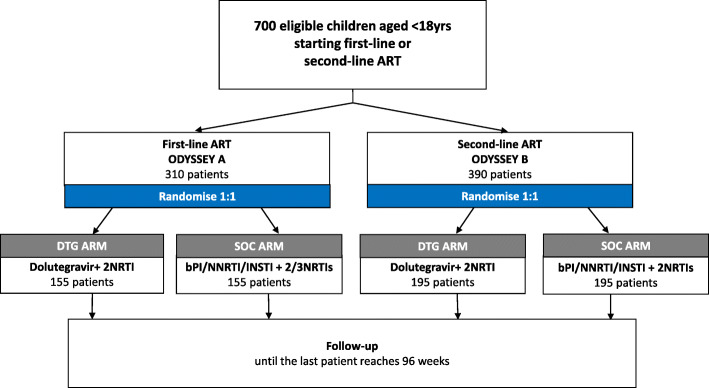
Trial Schema. Note: 85 additional children weighing 3 to < 14 kg were randomised to DTG versus SOC and will be followed up for 96 weeks**.** Abbreviations: ART = Antiretroviral Therapy; NRTI=Nucleos(t) ide reverse transcriptase inhibitors; bPI = boosted-Protease Inhibitors; NNRTI=Non-nucleoside reverse-transcriptase inhibitors; INSTI=Integrase Inhibitors

ODYSSEY is an open-label, multi-centre, randomised, non-inferiority, Phase II/III, 96-week basket trial, powered to evaluate DTG-based ART in 2 cohorts: ART-naïve children (ODYSSEY A) and children starting second-line (ODYSSEY B). Children (< 18 years) were recruited at sites located in Uganda, South Africa, Zimbabwe, Thailand, Portugal, Germany, Spain and the United Kingdom. A basket design allows for enrolment to occur to two (or more) cohorts simultaneously at the same clinical sites, thereby improving efficiency and minimizing time and costs [22]. At trial design we planned to randomise 700 children (310 in ODYSSEY A and 390 in ODYSSEY B) 1:1 to receive 2 NRTIs with DTG (DTG arm) or SOC (bPI-, NNRTI- or non-DTG INSTI-based ART; SOC arm) (Fig. 1). Randomisation of children was stratified by ODYSSEY A/ODYSSEY B, routine availability at site of resistance tests for children failing treatment, intended SOC (bPI/non-bPI ART) and intended NRTI backbone (both specified prior to randomisation). The randomisation list was prepared by the trial statistician using random permuted blocks and securely incorporated within the trial database. Randomisation of study participants was done via a web service accessed by site clinician or one of the coordinating trials units. Allocation was concealed until randomisation occurred after which allocation was open—i.e. clinicians and carers were aware of group assignment.

As a result of delays in dosing information for children < 14 kg, the trial was modified before recruitment closed to allow for the inclusion of ≥60 additional children weighing 3 to < 14 kg at enrolment (including a minimum of 20 children in each of the three lower weight-bands: 3 to < 6 kg, 6 to < 10 kg, 10 to < 14 kg) with participation in a PK sub-study for those randomised to the DTG arm. Minimum targets of 20 per weight-band were based on requiring ≥8 evaluable PK curves per weight band in the DTG arm and clinical opinion regarding numbers required < 14 kg to assess alongside the children ≥14 kg. Total enrolment was increased to allow for these additional children. Follow-up for the main trial population continued until the last ≥14 kg child recruited completed 96 weeks; each of the additional children < 14 kg is being followed-up for 96 weeks from randomisation. Including these extra children takes advantage of the opportunity ODYSSEY offers for a randomised comparison of DTG versus SOC in these younger/lighter children. Concurrent recruitment of children 3 to < 14 kg alongside the children weighing ≥14 kg was allowed for with randomisation stratified by < 14 kg and ≥ 14 kg to protect the main trial population ≥ 14 kg where randomisation was preserved. Randomisation of children < 14 kg was further stratified by weight-band (3 to < 6 kg, 6 to < 10 kg, 10 to < 14 kg) and ODYSSEY A/ODYSSEY B. Our design was planned to be robust if any/all of the lower weight-bands had to be dropped from the randomised comparison. Sub-study data were reviewed in children < 14 kg by the Independent Data Monitoring Committee (IDMC) (safety, efficacy and PK data) and by the Trial Steering Committee, ViiV Healthcare and the Trial Management Group (safety and PK data in DTG arm); had DTG been discontinued in any weight-band for safety concerns, PK concentration levels or lack of efficacy, we would have been able to drop children recruited in the respective weight-band (DTG and SOC participants) from our treatment comparisons. We are also able to analyse and report data on children enrolled weighing ≥14 kg while data are still accumulating in the children < 14 kg.

### Study population

Eligibility criteria for ODYSSEY have changed over time mostly to expand eligibility to younger/lighter children (Table 1). In the initial ODYSSEY protocol, children had to be over 6 years and weighing ≥20 kg to be eligible. Throughout the trial, eligibility in *both arms* was contingent on a confirmed DTG dose being available for a given age/weight-band (or eligibility for a PK sub-study). Delays in dosing information for children< 14 kg and fast enrolment of ART-naïve children ≥35 kg led to the decision to cap recruitment of ART naïve children ≥35 kg in July 2017. When we stopped screening children ≥35 kg for ODYSSEY A, 100/172 enrolled in A were ≥ 35 kg, with the majority (94/100) aged 12 years or over. Although no cap was included in the protocol, the Trial Management Group and oversight committees considered it important to increase numbers < 35 kg and to keep trial places for younger children.

**Table 1:** Participant Inclusion and Exclusion Criteria from Protocol Version 4.0 onwards

**Inclusion Criteria:**	
**All**	• Children ≥28 days and < 18 years weighing ≥3 kg with confirmed HIV-1 infection^a^
	• Parents/carers and children, where applicable, give informed written consent
	• Girls who have reached menses must have a negative pregnancy test at screening and randomisation and be willing to adhere to effective methods of contraception if sexually active
	• Children with co-infections who need to start antiretroviral therapy can be enrolled according to local/national guidelines
	• Parents/carers and children, where applicable, willing to adhere to a minimum of 96 weeks’ follow-up
**ODYSSEY A (First-line) only**	• Planning to start first-line antiretroviral therapy
**ODYSSEY B (Second-line) only**	• Planning to start second-line antiretroviral therapy defined as either: (i) switch of at least 2 antiretroviral therapy drugs due to treatment failure; or (ii) switch of only the third agent due to treatment failure where drug sensitivity tests show no mutations conferring nucleos(t) ide reverse transcriptase inhibitors resistance
	• Treated with only one previous antiretroviral therapy regimen. Single drug substitutions for toxicity, simplification, changes in national guidelines or drug availability are allowed
	• At least one nucleos(t) ide reverse transcriptase inhibitor with predicted preserved activity available for a background regimen
	• In settings where resistance tests are routinely available, at least one active nucleos(t) ide reverse transcriptase inhibitor from TDF/TAF, ABC or ZDV should have preserved activity based on cumulative results of resistance tests
	• In settings where resistance tests are not routinely available, children who are due to switch according to national guidelines should have at least one new nucleos(t) ide reverse transcriptase inhibitor predicted to be available from TDF/TAF, ABC or ZDV
	• Viral load ≥500 c/ml at screening visit or within 4 weeks prior to screening‡
**Exclusion Criteria:**	
All	• History or presence of known allergy or contraindications to dolutegravir
	• History or presence of known allergy or contraindications to proposed available nucleos(t) ide reverse transcriptase inhibitor backbone or proposed available standard-of-care third agent.
	• Alanine aminotransferase (ALT) ≥5 times the upper limit of normal (ULN), OR ALT ≥3xULN and bilirubin ≥2xULN
	• Patients with severe hepatic impairment or unstable liver disease (as defined by the presence of ascites, encephalopathy, coagulopathy, hypoalbuminaemia, oesophageal or gastric varices, or persistent jaundice), known biliary abnormalities (with the exception of Gilbert’s syndrome or asymptomatic gallstones)
	• Anticipated need for Hepatitis C virus therapy during the study
	• Pregnancy or breastfeeding
	• Evidence of lack of susceptibility to integrase inhibitors or more than a 2-week exposure to antiretrovirals of this class

^a^ Recruitment of ART naive children ≥35 kg was capped in Jul 2017. Recruitment opened in children ≥6 years and < 18 years weighing ≥20 kg (Protocol version 2.0). Protocol version 3.0 included recruitment of children ≥14 kg (with no age restriction) with a lead-in PK sub-study for children weighing 14 to < 20 kg (children weighing 14-15 kg had to be willing to enter the lead-in PK sub-study to enrol in the trial). Protocol version 4.0 onwards allows allowed for the recruitment of children ≥3 kg and ≥ 28 days, children weighing 3 to < 14 kg had to be willing to participate in a lead-in PK sub-study to enter the trial. Throughout the trial eligibility was based in both arms on availability of DTG dose given age/weight-band. ‡ Protocol version 3.0 onwards (viral load ≥1000 c/ml in protocol version 2.0)

### Treatment of patients

NRTI backbone and SOC third-agents are prescribed according to national guidelines. Planned treatment was recorded prior to randomisation which was stratified (for children ≥14 kg) by whether or not bPI would be offered as SOC and by NRTI backbone, to ensure treatment groups were balanced for these factors. In countries where drug resistance tests were routinely performed, it was recommended that at least one active NRTI was chosen. In countries where drug resistance tests were not routinely performed, it was recommended at least one NRTI was chosen with presumed preserved activity based on treatment history.

DTG dose by protocol version has changed over time (Table 2) and has been driven by new licensing information and emerging data from IMPAACT P1093 [24] and ODYSSEY PK sub-studies (see below).

**Table 2:** Dolutegravir dosing in odyssey main trial participants by protocol version^g^

WHO weight-band	FDA approval^a^		EMA approval^b^		ODYSSEY v2.0^a^	ODYSSEY v3.0	ODYSSEY v4.0	ODYSSEY v5.0/v6.0
					***(Sep 2016 onwards)***	***(Mar 2017 onwards)***	***(May 2018 onwards)***	***(May 2019/Feb 2020 onwards)***
3 to < 6 kg					–	–	–	–
6 to < 10 kg					–	–	–	–
10 to < 14 kg					–	–	–	–
14 to < 20 kg			20 mg^c^	Feb-2017	20 mg FCT^d^	20 mg FCT^d^	25 mg FCT → 25 mg DT^f^	25 mg DT
20 to <25 kg			25 mg	Feb-2017	25 mg FCT	25 mg FCT	25 mg FCT → 30 mg DT or 50 mg FCT^f^	50 mg FCT
25 to < 30 kg			25 mg	Feb-2017	25 mg FCT	25 mg FCT → 50 mg FCT^e^	25 mg FCT → 50 mg FCT^e^	50 mg FCT
30 to < 35 kg	35 mg	Jun-2016	35 mg	Feb-2017	35 mg FCT	35 mg FCT → 50 mg FCT^e^	35 mg FCT → 50 mg FCT^e^	50 mg FCT
35 to < 40 kg	35 mg	Jun-2016	35 mg	Feb-2017	35 mg FCT	35 mg FCT → 50 mg FCT^e^	35 mg FCT → 50 mg FCT^e^	50 mg FCT
≥40 kg	50 mg	Aug-2013	50 mg	Jan-2014	50 mg FCT	50 mg FCT	50 mg FCT	50 mg FCT

*Abbreviations*: *FCT* Film-coated tablets, *DT* Dispersible tablets, *v* Version

^a^ Approval in ≥12 years in Aug 2013, restriction removed Jun 2016

^b^ Approval in ≥12 years in Jan 2014, ≥6 years in Feb 2017

^c^ 20 mg FCT approved by EMA in children weighing 15 to < 20 kg only

^d^ From May 2017, ≥6 years, in children weighing 15 to < 20 kg only

^e^ From 1st of Apr 2018, after ethics notification, sites following protocol version 3.0 and above were recommended to increase the DTG dose of children 25 to < 40 kg to 50 mg FCT QD at their next scheduled study visit based on the results of the weight-band pharmacokinetic sub-study [23]

^f^ Protocol version 4.0 also allowed for the adjustment of weight-band dose based on findings from the ongoing weight-band pharmacokinetic sub-study with ethics notification

^g^ Protocol version 1.0 was not used

### Primary and secondary endpoints

The primary endpoint for the trial is virological or clinical failure by 96 weeks, which is defined as the first occurrence of any of the following: 1) insufficient virological response, 2) virological failure (two consecutive viral load measures ≥400 c/ml with the first at/after week 36), 3) new or recurrent AIDS defining event (WHO 4) or severe WHO 3 event, adjudicated by an independent blinded endpoint review committee or 4) all-cause death. The primary comparison is DTG vs SOC in the combined trial population (first-line and second-line children combined); subpopulation analyses (ODYSSEY A and ODYSSEY B) are key secondary comparisons. The components of the primary composite endpoint (virological failure and clinical failure) are included as secondary endpoints in addition to other key outcomes (Table 3).

**Table 3:** Primary and secondary outcome measures in odyssey

**Primary Efficacy Outcome**	
Difference in proportion with clinical or virological failure by 96 weeks, defined as the first occurrence of any of the following components: 1. Insufficient virological response defined as <1 log10 drop at week 24 (or viral load ≥50c/mL at week 24 in a participant with viral load < 500c/mL at baseline) and switch to second/third line antiretroviral therapy for treatment failure 2. Virological failure (defined as a viral load of greater than or equal to 400 copies/mL at or after week 36 confirmed by the next visit) 3. New or recurrent AIDS defining event (WHO 4) or severe WHO 3 event, confirmed by the Endpoint Review Committee (see Appendix) 4. All-cause death	
**Secondary efficacy outcomes**	
Difference in proportion with clinical or virological failure (as defined above) by 48 weeks	
Time to any new or recurrent AIDS defining event (WHO 4) or severe WHO 3 events, confirmed by the Endpoint Review Committee	
Proportion of children with Viral Load < 50 c/ml at 48 and 96 weeks	
Proportion of children with Viral Load < 400 c/ml at 48 and 96 weeks	
Rate of HIV-associated events (WHO 4 and severe WHO 3) and death over 96 weeks	
Change in CD4 count and percentage and CD4/CD8 ratio from baseline to weeks 48 and 96	
Proportion developing new resistance mutations	
**Secondary safety outcomes**	
Change in total cholesterol, triglycerides and lipid fractions (high-density lipoproteins**,** low-density lipoproteins) from baseline to weeks 48 and 96 (change in total cholesterol from baseline to week 96 will be used to formally assess superiority of dolutegravir-based regimen vs. standard-of-care)	
Incidence of serious adverse events	
Incidence of new clinical and laboratory grade 3 and 4 adverse events	
Incidence of adverse events (of any grade) leading to treatment modification	
**Other secondary outcomes**	
Quality of life	
Adherence and acceptability	

### Sample size calculations

A non-inferiority margin of 10–12% was chosen, in accordance with previous HIV treatment trials [25, 26]. Assuming a failure rate of 18% overall (first-line and second-line children combined) in the DTG arm and SOC arm by 96 weeks and allowing for 10% loss to follow-up, 700 children provide 90% power to exclude (at two-sided 5% significance level) a difference of more than 10% in the primary outcome between the two arms. Enrolling 310 first-line ART and 390 s-line ART children provide 80% power to exclude a difference of more than 12% between the DTG and SOC arms in both subgroups separately, assuming a failure rate of 15% in ART-naïve (based on previous trials [27]) and 20% in ART-experienced children with 10% loss to follow-up.

### Sub-studies

#### PK sub-studies

Weight-band PK sub-studies were introduced in Protocol version 3.0 (March 2017) with the aim of simplifying doses (using WHO weight-bands with no age restrictions) and using DTG formulations planned for manufacturing by generic companies.

In the first sub-study, PK and safety data were collected on 33 children weighing 14 to < 25 kg on DTG 25 mg FCT (Table 4). Exposures were low compared to reference data in adults [28].

**Table 4:** Dolutegravir dosing in odyssey pk sub-study participants by protocol version

WHO weight-band	Initiated under Protocol v3.0	Initiated under Protocol v4.0
3 to < 6 kg	–	Single PK Curve on 5 mg or 10 mg DT^a^
6 to < 10 kg	–	Single PK Curve on 15 mg DT
10 to < 14 kg	–	Single PK Curve on 20 mg DT
14 to < 20 kg	Single PK Curve on 25 mg FCT	Single PK Curve on 25 mg DT^b^
20 to <25 kg	Single PK Curve on 25 mg FCT	Single PK Curve on 30 mg DT or 50 mg FCT^b^
25 to < 30 kg	Cross-over PK with one curve on 25 mg FCT and second curve on 50 mg FCT	–
30 to < 35 kg	Cross-over PK with one curve on 35 mg FCT and second curve on 50 mg FCT	–
35 to < 40 kg	Cross-over PK with one curve on 35 mg FCT and second curve on 50 mg FCT	–
≥40 kg	–	–

*Abbreviations*: *FCT* Film-coated tablets, *DT* Dispersible tablets, *v* Version, *PK* Pharmacokinetic

^a^DTG dose for infants 3- < 6 kg is dependent on age initially. Infants < 6 months of age receive DTG 5 mg QD while infants ≥6 months of age receive DTG 10 mg QD, both as dispersible tablets

^b^Preference given to enrolment of children who completed PK on 25 mg FCT initiated under protocol version 3.0

In the second sub-study, 28 children weighing 25 to < 40 kg had PK on their initial dose (25 mg or 35 mg FCT); they then switched to the adult 50 mg FCT dose and underwent further PK and safety monitoring (Table 4). Based on the results of this sub-study [23] and following ethics approval, all children in the main trial weighing ≥25 kg moved to the adult 50 mg FCT dose.

Based on the results of the first two sub-studies, children weighing 14 to < 20 kg were changed to DTG 25 mg dispersible tablets (5x5mg DT which have higher bioavailability than FCTs), and children weighing 20 to < 25 mg were changed to DTG 30 mg DT (6x5mg DT) or DTG 50 mg FCT, with further PK and safety monitoring. Data on children weighing 20 to < 25 mg taking the adult DTG 50 mg FCT also demonstrated appropriate PK profiles [29].

Following review of ODYSSEY PK sub-study results by the Pediatric Antiretroviral Working Group (PAWG) of the WHO, WHO paediatric dosing guidelines have been updated to allow children 20 to < 40 kg to receive the adult 50 mg FCT dose [19].

Recruitment of children weighing 3 to < 14 kg was initiated under protocol version 4.0 and children randomised to DTG completed PK assessments [30].

A TB-PK sub-study with a within-child cross-over design is also evaluating the PK of DTG co-administered with rifampicin in HIV/tuberculosis (TB) co-infected children. Children on DTG diagnosed with TB start on rifampicin (RIF) and receive BID DTG (doubled daily dose) for the duration of TB treatment, and 2 weeks following. The first PK is undertaken at the end of TB treatment; followed by a second PK on QD DTG (once daily) ≥4 weeks after RIF is stopped, to allow for a wash-out period [31].

**An immunology/virology sub-study** will explore mechanisms of CD4 reconstitution, immune activation, HIV reservoir and low-level viral replication in the DTG and SOC arms.

**A qualitative sub-study** is investigating how best to support young people to maintain optimal adherence to second-line treatment, using interviews and focus groups with a small proportion of participants and carers in ODYSSEY B [32]. Young people and caregivers’ understanding of the DTG pregnancy safety alert have also been explored.

**A folate and vitamin B12 sub-study** at Ugandan sites was added in protocol v5.0 following the concerns raised around an increased risk of NTDs in babies born to mothers on DTG at conception [18]. Red cell folate and vitamin B12 levels will be assayed in samples prospectively collected at week 96 or later and compared between trial arms; change in plasma folate levels between week 4 and baseline (using routinely collected stored samples) will also be compared between arms.

In addition to the sub-studies, **Youth Trial Boards** in Uganda, Zimbabwe, South Africa and the UK have been developed alongside ODYSSEY to allow for meaningful engagement and participation of adolescent patient representatives in paediatric clinical trials. To date, they have had input into patient information sheets, have developed tools for explaining the risks of DTG in pregnancy to young people, and have participated in the development of a WHO toolkit and WHO webinars on youth participation in research.

### Study procedures

All participants are seen at screening, enrolment, week 4, 12 and every 12 weeks to the end of follow-up. Every visit includes a clinical assessment including height, weight (and adjustment of drug doses accordingly), change in HIV disease stage, clinical events, adverse events and an adherence assessment (Table 5). In protocol version 3.0, questionnaires assessing mood and sleep symptoms were added. CD4, CD8, biochemistry and haematology laboratory tests are done at baseline, 4 weeks, 24 weeks and then 24-weekly, with lipids and glucose measured at baseline and 48-weekly. At sites where viral load is routinely measured every 12–16 weeks, real-time viral loads are measured at screening, week 4, 12, 24 and then every 12 weeks. At other sites, a real-time viral load is measured at screening (unless one is available from the last 4 weeks) to confirm eligibility in ODYSSEY B participants; otherwise viral loads are done according to local routine practice, and plasma samples stored at the above time-points for retrospective testing. Retrospective viral load testing is done in batches, and results are not used for patient management; this ensures generalisability of the trial results to LMIC settings where viral load is measured only 6 or 12-monthly according to WHO [33] and national guidelines. All viral load results are reviewed during the trial by the IDMC and we aim to have results of batched testing up-to-date for each IDMC review.

**Table 5:** Clinical assessments undertaken and frequency of testing

Clinical Assessment	Frequency of Assessment
History and clinical assessment including height, weight (and adjustment of drug doses accordingly) and mid upper arm circumference, change in HIV disease stage, clinical events and presence of adverse events.	Every study visit
Tanner scale (children aged 8 or over)	Baseline, then every 24 weeks and at the end of study visit
Lipodystrophy assessment including at selected sites bioelectrical impedance analysis.	Baseline, then every 48 weeks and at the end of study visit
HIV-1 RNA viral load	Every study visit as per local practice. In ODYSSEY B viral load must be measured at screening unless the viral load has been done within 4 weeks prior to screening. Stored plasma samples will be used to examined viral load for study visits where viral load is not routinely done.
T cell lymphocyte subsets including CD4 and CD8 percentage and absolute, total lymphocyte count	Baseline, 4, 12, 24, then 24-weekly until week 96 and at the end of study visit
Biochemistry including creatinine, bilirubin, alanine aminotransferase (ALT), and optionally aspartate aminotransferase (AST)	Baseline, 4, 24, then 24-weekly until week 96 and at the end of study visit
Haematology including haemoglobin, mean corpuscular volume (MCV), white blood cell count, lymphocytes, neutrophils, and platelets	Baseline, 4, 24, then 24-weekly until week 96 and at the end of study visit
Lipids/glucose including fasted triglycerides, cholesterol (total, high-density lipoproteins, low-density lipoproteins), and glucose	Baseline, 48, 96 weeks, then every 48 weeks and at the end of study visit
Bone profile including calcium, phosphate, alkaline phosphatase	Optional for all sites at baseline, 48, 96 weeks, then every 48 weeks and at the end of study visit
Urine dipstick for protein and glucose	Baseline, 48, 96 weeks then every 48 weeks and at the end of study visit
Quality of Life questionnaire	Baseline, 12, 24, 48, 96 weeks then every 48 weeks and at the end of study visit
Pregnancy test (for all females of childbearing potential)	This was originally at baseline and then every 24 weeks but following the pregnancy alert in DTG in May 2018 this was updated to every study visit (see Section entitled ‘Pregnancy Alert’)
Plasma storage for retrospective HIV-1 RNA viral load, resistance testing where not routinely available locally and sub-study assays	Every visit
Peripheral blood mononuclear cell storage at selected sites for immunology/virology sub-study	At selected sites for baseline, 12, 48, and 96 weeks
Adherence questionnaire	Every visit
Acceptability, sleep & mood questionnaire^a^	Baseline, 4, 12, 24, 48, 72, 96 weeks, then every 24 weeks and at the end of study visit. Also acceptability should be completed if treatment failure has occurred or the antiretroviral therapy regimen changed.

^a^ Sleep and mood questions including from protocol version 3.0 onwards

### Study organisation, governance and ethics

The sponsor of the trial is the Paediatric European Network for Treatment of AIDS (PENTA) Foundation (https://penta-id.org/). Management of the trial is delegated to the clinical trials units: Medical Research Council Clinical Trials Unit at UCL (MRC CTU at UCL), French National Institute for Health and Medical Research-French National Agency for Research on AIDS and Viral Hepatitis (INSERM-ANRS) and PHPT-UMI 174 in Thailand. The trial was submitted for approval by Research Ethics Committees/Institutional Review Boards and by all required regulatory authorities in each of the participating countries.

### Statistical analysis plan

The primary analysis will take place after the last participant recruited weighing ≥14 kg has completed 96 weeks and all children ≥14 kg have had a study closeout visit and will only include data in children recruited weighing ≥14 kg. The cumulative probability of clinical or virological failure by week 96 will be compared in the DTG and SOC arms, using the intention-to-treat population, estimated by the Kaplan-Meier Method adjusting for stratification factors. If the upper limit of the confidence interval for the difference between arms is < 10%, DTG-based ART will be considered non-inferior to SOC.

The primary endpoint includes confirmed HIV RNA ≥ 400 c/ml at or after 36 weeks. Since sites with frequent real-time viral load testing are likely to confirm failure more quickly than sites testing 6-monthly or annually, to ensure consistency for this endpoint the date of next scheduled visit following the first HIV RNA ≥400 (using schedule from randomisation) will be used for time to first confirmed viral load measurement ≥400c/ml (primary endpoint failure date), rather than the date of the confirmatory measure. Sensitivity analyses will include defining failure at first of two consecutive HIV RNA ≥ 400 c/ml at or after 36 weeks.

A small number of participants were randomised with minor ineligibility reasons and were retained in the trial. A secondary analysis will be done in the per protocol population excluding participants who did not meet the eligibility criteria and censoring follow-up at any change to the third agent (i.e. a change in either an INSTI, a PI, or an NNRTI, including adding an additional third agent or stopping treatment for > 31 days) for toxicity, failure or pregnancy/planned pregnancy.

Participants with VL < 50 (or < 400) c/ml at 48 and 96 weeks will be compared between arms based on (i) crude proportions and (ii) using the FDA snapshot algorithm. Change in total cholesterol, triglycerides and lipid fractions (LDL, HDL) from baseline to weeks 48 and 96 will be analysed using analysis of covariance to compare the change in mean level between the two arms, adjusting for baseline value and stratification factors. Other endpoints will be assessed using appropriate statistical methods.

There will be a secondary analysis of primary and secondary endpoints in children recruited weighing < 14 kg when this group has completed 96 weeks follow-up. Bayesian estimation will be used to report the primary endpoint, incorporating evidence obtained from participants enrolled ≥14 kg as a prior distribution. Clinical opinion has been elicited on the relative weight to allocate to the prior distribution [34]. The Bayesian analysis will be reported alongside frequentist analyses of the < 14 kg participants and of the whole trial population (< and ≥ 14 kg).

## Results

### Challenges in study implementation- changing landscape of DTG dosing

As described above, eligibility to ODYSSEY has expanded since the trial began to include younger/lighter children and DTG doses have been tested and modified within weight-bands, with protocol amendments, based on the PK sub-study results [23, 29] and with IDMC, TSC and ethics approval. This has increased the complexity of the trial for trial teams, particularly at PK sites. Drug supply management has also been challenging, to ensure correct DTG formulations are available at clinical centres in a timely manner.

The impact of using different DTG dosing within the same weight-band on the main trial comparison has been considered. We have taken the view that the trial will be a pragmatic comparison of DTG vs SOC for clinical or virological failure, and consider that it is more valuable to collect follow-up information on the doses likely to be licensed and used, particularly in low-resource settings.

### Challenges in study implementation- pregnancy alert

The following measures were in place for girls of childbearing age prior to the DTG safety alert reporting a possible association between becoming pregnant on DTG and an increased risk of neural tube defect in the infant [15]:

- Eligibility contingent on negative pregnancy test and adherence to effective methods of contraception (Table 1)
- A pregnancy test every 24 weeks
- Pregnancies in trial participants reported, and outcomes followed-up
- Clinician discretion with respect to continuing DTG during pregnancy, with advice to consider alternative ART.

Following the safety alert, the trial team took the following actions:

- Participants were notified of this potential safety risk, prioritising those ≥15 years.
- Patient information sheets and consent forms were updated
- Availability of contraception and contraceptive counselling at each site was reviewed, and strengthened if necessary. Case report forms were revised to collect information about use of contraceptives.
- Participants in the DTG arm who have a positive pregnancy test within 8 weeks of their last menstrual period or who wish to become pregnant are now advised to switch to a non-DTG ART regimen in consultation with their clinician.
- The frequency of pregnancy testing for girls of childbearing age was increased to every (12-weekly) follow-up visit.
- Collection of data on use of folate supplements (advised during pregnancy) was strengthened.
- Youth Trial Board members created adolescent-friendly information on the safety alert, which can be provided to trial participants.

### Post-trial access to DTG

Adult DTG (50 mg FCT) has been registered in all participating countries and is widely available in Europe and Africa. DTG DT formulations are unlikely to be available by the end of the trial. Protocol v6.0 includes extended observational follow-up for children in Africa and Thailand in both trial arms for a further 3 years; children on DTG at the end of the trial who continue in follow-up will be provided with DTG DT and (in Thailand) 50 mg FCT through ViiV Healthcare as necessary.

### Current status of the trial

Between 20th September 2016 and 22nd June 2018, 707 children ≥14 kg were enrolled, including 311 ART-naïve children and 396 children starting second-line; enrolment was then closed to children ≥14 kg. Table 6 describes the baseline characteristics of children enrolled ≥14 kg: 621 (88%) have been enrolled in Africa; median (range) age was 12.2 [2.9–18.0]; 128 (18%) had WHO stage 3 and 60 (8%) WHO stage 4 disease; the majority were vertically infected. SOC is primarily efavirenz-based for ART-naïve children and lopinavir/ritonavir-based for ART-experienced children with abacavir+lamivudine or tenofovir+lamivudine/emtricitabine. Children recruited ≥14 kg all reached 96 weeks follow-up by end April 2020, with primary results expected by early 2021. Recruitment of children weighing 3 to < 14 kg started 5th July 2018 and completed 26th August 2019; 85 children were recruited, all in African sites, including 23 weighing 3 to < 6 kg, 39 weighing 6 to < 10 kg and 23 weighing 10 to < 14 kg at enrolment.

**Table 6:** Baseline characteristics of odyssey participants ≥14 kg at randomisation

		ODYSSEY A		ODYSSEY B		Total	
		(First-line)		(Second-line)			
Participants randomised ≥14 kg		311		396		707	
Country/region							
Europe		18	(6%)	7	(2%)	25	(4%)
South Africa		76	(24%)	67	(17%)	143	(20%)
Thailand		50	(16%)	11	(3%)	61	(9%)
Uganda		99	(32%)	232	(59%)	331	(47%)
Zimbabwe		68	(22%)	79	(20%)	147	(21%)
Sex							
Male		147	(47%)	215	(54%)	362	(51%)
Female		164	(53%)	181	(46%)	345	(49%)
Age, years							
2 to < 6		12	(4%)	14	(4%)	26	(4%)
6 to < 12		155	(50%)	161	(41%)	316	(45%)
12 to < 18		144	(46%)	221	(56%)	365	(52%)
Weight, kg							
14 to < 20		38	(12%)	44	(11%)	82	(12%)
20 to < 25		62	(20%)	73	(18%)	135	(19%)
25 to < 30		58	(19%)	59	(15%)	117	(17%)
30 to < 35		33	(11%)	56	(14%)	89	(13%)
35 to < 40 kg		22	(7%)	39	(10%)	61	(9%)
≥40 kg		98	(31%)	125	(32%)	223	(31%)
BMI-for-Age Z-Score^a^							
<−3		12	(4%)	21	(5%)	33	(5%)
−3 to < −2		24	(8%)	24	(6%)	48	(7%)
−2 to < 0		178	(57%)	257	(65%)	435	(62%)
≥0		97	(31%)	94	(24%)	191	(27%)
Mode of infection							
Mother-to-child		240	(77%)	365	(92%)	605	(86%)
Blood product		1	(<1%)	2	(1%)	3	(<1%)
Sexual contact		36	(12%)	3	(1%)	39	(6%)
Unknown		32	(10%)	26	(7%)	58	(8%)
Other^d^		2	(1%)	0	(0%)	2	(<1%)
CD4, cells/mm3^b^							
< 100		56	(18%)	54	(14%)	110	(16%)
100 to < 200		20	(6%)	29	(7%)	49	(7%)
200 to < 500		103	(33%)	128	(32%)	231	(33%)
500 to < 1000		99	(32%)	130	(33%)	229	(32%)
≥1000		33	(11%)	55	(14%)	88	(13%)
CD4, %							
< 15		110	(35%)	119	(30%)	229	(32%)
15 to < 25		100	(32%)	104	(26%)	204	(29%)
≥25		101	(32%)	173	(44%)	274	(39%)
Median CD4 (IQR), cells/mm3^b^		436	(210–660)	482	(243–752)	459	(228–704)
Viral load, copies/mL^b^							
< 1000		25	(8%)	6	(2%)	31	(4%)
1000 to < 10,000		62	(20%)	122	(31%)	184	(26%)
10,000 to < 50,000		76	(24%)	151	(38%)	227	(32%)
50,000 to < 100,000		44	(14%)	44	(11%)	88	(13%)
≥100,000		101	(33%)	73	(18%)	174	(25%)
Median Log10 Viral load (IQR), copies/mL^b^		4.6	(3.9–5.1)	4.3	(3.8–4.8)	4.4	(3.9–5.0)
Clinical (WHO) Staging							
I		127	(41%)	143	(36%)	270	(38%)
II		113	(36%)	135	(34%)	248	(35%)
III		47	(15%)	81	(21%)	128	(18%)
IV		23	(8%)	37	(9%)	60	(8%)
Previous ART Class Exposure^c^							
NRTI/NNRTI				382	(96%)		
NRTI/NNRTI/PIs				4	(1%)		
NRTI/PIs				10	(3%)		
Median cumulative ART exposure (IQR), years^c^				5.5	(3.5–8.1)		

Percentages are of non-missing values. *Abbreviations*: *BMI* Body Mass Index, *ART* Antiretroviral Therapy, *NRTI* Nucleos(t) ide reverse transcriptase inhibitors, *PI* Protease Inhibitors, *NNRTI* Non-nucleoside reverse-transcriptase inhibitors, *IQR* Interquartile Range

^a^Assessed using World Health Organization (WHO) 2007 STATA macro package [35].

^b^Mean of results at screening and randomisation if both are available

^c^Excluding any PMTCT exposure

^d^ 1 injecting drug use; 1 possible transmission father-to-child

## Discussions

The ODYSSEY trial is an ambitious trial which has recruited HIV-infected children across 29 sites in 8 countries to evaluate DTG-based ART versus SOC. Recruitment of ≥700 children was completed ahead of target and an additional 85 children < 14 kg were recruited over 13 months. ODYSSEY employs a basket design to include ART-naïve and ART-experienced children, and, although the primary comparison will be in all children, the trial is powered to evaluate non-inferiority of DTG vs SOC in both sub-groups. To ensure relevance of findings across sub-Saharan Africa, where most HIV-infected children live, national guidelines are adhered to for real-time viral load testing and retrospective viral load testing is used to determine the trial’s virological outcomes.

When ODYSSEY was planned it was assumed that DTG dosing within the trial would follow recommendations arising from the IMPAACT P1093 paediatric DTG dosing study. In reality, dosing information became available slower than expected, while recruitment to ODYSSEY was faster than expected. In response, ODYSSEY included several nested PK sub-studies to allow recruitment of young children in the three lower weight bands. Pragmatic (WHO weight band-based) paediatric dosing using minimum formulations was assessed across the PK substudies aiming to simplify future procurement of paediatric formulations. Evaluation of adult DTG dosing down to children weighing 20 kg was incorporated, with the goal of enabling rapid access of DTG for the majority of children and easier implementation within LMICs national treatment programmes.

The advantages of nested PK in a larger trial translate into faster recruitment into PK studies and the generation of long-term safety data. Already, PK and safety data from ODYSSEY on the use of the adult DTG 50 mg FCT in children weighing ≥20 kg have been incorporated into WHO dosing guidelines [19]. WHO has predicted that this approach of conducting a parallel dose finding study (IMPAACT P1093) with a long term efficacy, safety and simplified dosing study (ODYSSEY) will likely result in ~ 500,000 children that can start or switch to DTG 3 years earlier than would otherwise have been possible [36].

Recruitment of children < 14 kg will provide valuable efficacy and safety data in this group. This was allowed for in a protocol amendment, with randomisation stratified to protect the main trial. Data from the weight-band PK sub-studies were included as part of FDA and EMA licensing applications for TIVICAY™ submitted by ViiV Healthcare and GlaxoSmithKline; with FDA approval for paediatric DTG received in June 2020 and EMA approval expected early 2021.

## Conclusions

As identified by the WHO and the Collaborative Initiative for Pediatric HIV Education and Research (CIPHER), we must urgently accelerate the paediatric HIV response with evidence-based interventions to maximize short- and long-term outcomes in children. Through responsive and innovative design features, the ODYSSEY trial is contributing to this global research agenda, by efficiently answering multiple research questions around use of DTG in paediatric HIV care.

## Data Availability

The ODYSSEY trial data are held at MRC CTU at UCL, which encourages optimal use of data by employing a controlled access approach to data sharing (http://www.ctu.mrc.ac.uk/our_research/datasharing/). All requests for data are considered and can be initiated by contacting mrcctu.trial-odyssey@ucl.ac.uk or through the URL: https://www.ctu.mrc.ac.uk/our-research/other-research-policy/data-sharing/application-process/

## Appendix

**Table 7:** Severe who clinical stage 3 for the odyssey trial

Children (< 15 years of age)	Adolescents (≥15 years of age)
• Unexplained moderate malnutrition or wasting not adequately responding to standard therapy • Persistent oral candidiasis (after first 6–8 weeks of life) • Symptomatic lymphoid interstitial pneumonitis • Chronic HIV-associated lung disease including bronchiectasis	• Unexplained severe weight loss (> 10% of presumed or measured body weight) • Persistent oral candidiasis

Reference: World Health Organization. WHO case definitions of HIV for surveillance, and revised clinical staging and immunological classification of HIV related disease in adults and children. Geneva: World Health Organization; 2007

## Abbreviations

ART: Antiretroviral Therapy
bPI: Boosted protease inhibitor
CHAI: Clinton Health Access Initiative
CIPHER: Collaborative Initiative for Pediatric HIV Education and Research
DTs: Dispersible tablets
DTG: Dolutegravir
EMA: European Medicines Agency
FCT: Film coated tablet
HIV: Human immunodeficiency virus
IDMC: Independent Data Monitoring Committee
LMICs: Low- and middle-income countries
NTD: Neural tube defects
NNRTI: Non-nucleoside reverse transcriptase inhibitor
NRTIs: Nucleos(t)ides
PENTA: Paediatric European Network for Treatment of AIDS
PAWG: Pediatric Antiretroviral Working Group
PK: Pharmacokinetic
PEPFAR: President’s Emergency Plan for AIDS Relief
RIF: Rifampicin
SOC: Standard-of-care
TB: Tuberculosis
FDA: US Food and Drug Administration
WHO: World Health Organisation
